# Whey Protein Biopolymer Coatings for Sustainable Preservation of Potato Quality During Storage

**DOI:** 10.3390/polym17212860

**Published:** 2025-10-27

**Authors:** Hadeel Obeidat, Haneen Tarawneh, Samar Shawaqfeh, Rawan Al-Jaloudi, Yousef H. Tawalbeh, Deia Tawalbeh, Sarah Jaradat, Jomanah ALbtoosh, Dima Alkadri, Nawal Alsakarneh, Hala K. Nawaiseh, Moroug Zyadeh, Esma Foufou, Motasem AL-Masad, Nizar Alrabadi

**Affiliations:** 1 Department of Nutrition and Dietetics, Irbid National University, Irbid 21110, Jordan; 2 Department of Food Science and Nutrition, Jerash University, Jerash 26250, Jordan; h.tarawneh@jpu.edu.jo (H.T.); y_tawalbeh@ymail.com (Y.H.T.); d.al-tawalbeh@jpu.edu.jo (D.T.); d.alqaderi@jpu.edu.jo (D.A.); rabadinizar@yahoo.com (N.A.); 3 Department of Plant Production and Protection, Jerash University, Jerash 26250, Jordan; s.shawaqfeh@jpu.edu.jo; 4 Department of Allied Medical Sciences, Zarqa University College, Al-Balqa Applied University, Zarqa 13110, Jordan; 5 Department of Nutrition and Food Technology, The University of Jordan, Amman 11941, Jordan; sa.jaradat@ju.edu.jo (S.J.); ha.nawaiseh@ju.edu.jo (H.K.N.); 6 Department of Land, Water and Environment, The University of Jordan, Amman 11941, Jordan; jomanabtoosh@gmail.com; 7 Department of Nutrition and Food Processing, Al Huson University College, Al-Balqa Applied University, Irbid 21510, Jordan; nawal.sakarneh@bau.edu.jo; 8 Department of Plant Production-Smart and Sustainable Agriculture, Ajloun National University, Ajloun 26810, Jordan; morougzeadeh@yahoo.com; 9 Department of Food Technology, University of Constantine 1, Constantine 21000, Algeria; asma.foufou@umc.edu.dz; 10 Department of Animal Production and Protection, Jerash University, Jerash 26250, Jordan; mmamasad@hotmail.com

**Keywords:** whey protein concentrate, edible coatings, potato quality, chitosan, firmness, total soluble solids, dry matter, storage, biopolymer

## Abstract

Potato is a widely consumed staple crop prone to postharvest deterioration and quality loss. Biodegradable edible coatings offer an eco-friendly alternative to conventional packaging for extending shelf life. This study evaluated the effectiveness of whey protein concentrate (WPC) based coatings, with and without chitosan, in maintaining potato quality under different storage conditions and durations. Tubers were treated with WPC coating (WC1) or WPC–chitosan coating with additives (WC2) and stored at room temperature (RT, 24 °C), refrigeration (RF, 4 °C), or incubator (IC, 20 °C) for up to 48 days. Dry matter (DM), firmness (FR), and total soluble solids (TSS) were determined every 8 days. DM ranged between 17.3–20.7%, FR between 5.6–8.1 N, and TSS between 3.4–5.3 °Brix. Storage period (SP) had the strongest influence, with DM peaking after 16–24 days, FR gradually decreasing, and TSS dropping sharply after 32 days. Coating did not significantly affect DM, but WC2 improved FR retention while slightly lowering TSS. RF best preserved FR and TSS, whereas RT and IC accelerated quality loss. Overall, WPC-based coatings, particularly WC2, provide a biodegradable and effective strategy to reduce postharvest losses, maintain potato quality, and support sustainable food preservation.

## 1. Introduction

Global population growth and increasing food demand continue to challenge agricultural systems, underscoring the urgent need for sustainable production and preservation strategies [1]. Climate change, soil degradation, and postharvest losses intensify these challenges, emphasizing approaches that enhance resource efficiency while reducing waste. Sustainable agriculture integrates productivity with environmental protection by optimizing resource use and promoting long-term resilience [2]. It is estimated that approximately one-third of all food produced for human consumption is lost annually, drawing attention to the necessity of environmentally friendly preservation technologies [3]. Among the promising solutions, edible coatings made from natural biopolymers provide an effective, low-impact means to reduce moisture loss, delay respiration, and maintain sensory and nutritional quality [4].

Whey protein-based coatings are notable for their excellent gas-barrier properties, biodegradability, and mechanical strength [5]. Whey protein, composed mainly of β-lactoglobulin and α-lactalbumin, forms cohesive films through intermolecular hydrogen bonds and disulfide linkages, resulting in networks with low oxygen permeability and good elasticity [6]. These films can extend the storage life of perishable foods by protecting them against oxidative deterioration and moisture migration [7]. Furthermore, whey protein films can serve as carriers for antimicrobial or antioxidant agents, offering multifunctional benefits in food preservation [5,6].

Chitosan, a naturally occurring cationic polysaccharide derived from chitin, has also gained prominence in edible coating formulations. Structurally, it consists of β-(1→4)-linked D-glucosamine and N-acetyl-D-glucosamine units [8]. The protonation of its amino groups in acidic media imparts a positive charge that facilitates electrostatic interaction with negatively charged microbial membranes, leading to cell permeability disruption and death [9]. The physicochemical properties of chitosan—particularly its degree of deacetylation and molecular weight—strongly influence solubility, viscosity, and antimicrobial activity. A higher degree of deacetylation enhances ionic reactivity and solubility, whereas molecular weight determines mechanical resistance and flexibility [8]. Additionally, hydrogen bonding and crystallinity regulate permeability and film cohesion, contributing to its functional performance [10].

When combined with whey protein, chitosan improves the coating’s structure and functionality through electrostatic and hydrogen-bond interactions between protonated amino groups and protein carboxyl sites. These interactions generate uniform, compact matrices with enhanced tensile properties, lower water vapor transmission, and improved elasticity [11]. The antimicrobial characteristics of chitosan complement the gas-barrier and moisture-resistant behavior of whey protein, collectively preventing microbial growth and quality loss during storage [12]. The synergistic effect of these two biopolymers provides a multifunctional barrier that is mechanical, biochemical, and microbiological in nature, improving the longevity and safety of fresh produce [13]. Several studies have shown that whey protein concentrate (WPC) coatings effectively maintain the postharvest quality of fruits and vegetables by reducing weight loss and respiration rates and by retaining dry matter (DM), firmness (FR), and total soluble solids (TSS) [14]. The protective network formed by WPC stabilizes cellular moisture and modulates gas exchange, thereby maintaining FR and TSS stability during prolonged storage [15,16]. The inclusion of chitosan enhances these effects through additional moisture retention and antimicrobial protection, leading to better textural and compositional stability under varying storage conditions [17].

In Jordan, achieving agricultural sustainability requires integrating productivity gains with technological innovations that minimize postharvest losses. Financial support, technology adoption, and youth participation are crucial elements in advancing this sector [18,19,20,21]. However, losses in potatoes remain substantial due to the lack of proper storage infrastructure and cold-chain facilities [22]. The use of biodegradable coatings based on WPC and chitosan provides a feasible and sustainable approach to improve potato storage stability, maintain physicochemical quality, and reduce national food waste.

Therefore, this study investigates the performance of WPC-based edible coatings, with and without chitosan, in preserving potato quality. The effects of treatment and storage period (SP) on DM, FR, and TSS were analyzed under different storage conditions to determine how the physicochemical properties of chitosan influence coating behavior. By clarifying the relationship between polymer interactions and postharvest quality maintenance, this study contributes to developing sustainable, biodegradable strategies for extending shelf life and enhancing storage performance.

## 2. Materials and Methods

### 2.1. Plant Material

Potatoes (*Solanum tuberosum* L.) of uniform size were obtained from the central vegetable market in Irbid, Jordan. Tubers were carefully inspected, and abnormal, rotten, or physically damaged samples were discarded.

### 2.2. Edible Coating Preparation

Two edible coatings were formulated:

WPC coating (WC1): 50 g of whey protein concentrate (WPC; Gainland Chemical Company, Dukinfield, UK) was dissolved in 1000 mL distilled water and heated in a water bath at 80–90 °C for 30 min to induce protein denaturation, with continuous stirring using a magnetic stirrer (MS-H–S, Dragon Lab, USA).

WPC–chitosan coating with additives (WC2):

A chitosan stock was first prepared at 1% (*w*/*v*) in 1% (*v*/*v*) acetic acid: 5 g high-molecular-weight chitosan (degree of deacetylation = 99%, average molecular weight = 100 kDa; Sigma-Aldrich, St. Louis, MO, USA) was slowly sprinkled into 500 mL of 1% acetic acid under vigorous stirring. The mixture was stirred overnight at room temperature until visually clear. The solution was then coarsely filtered (≈100 µm mesh) to remove undissolved fines and de-gassed briefly. While stirring vigorously, the chitosan solution was titrated dropwise with 1 M NaOH to pH 5.6; base was added along the vortex wall while the beaker sat in a cool-water bath to avoid local deprotonation and precipitation. After pH adjustment, 5 mL glycerol (plasticizer), 1 mL Tween-80 (emulsifier; TEDIA Firland, Rocky Hill, Germany), and 1 mL coconut oil (antimicrobial) were added sequentially under continuous stirring (15–20 min). The conditioned chitosan phase was then slowly streamed into the warm WPC solution (from WC1) under moderate stirring and homogenized gently for 5 min (magnetic stirrer) to obtain a uniform emulsion. The resulting WC2 showed no visible phase separation for 1–2 h prior to application; a slight viscosity increase over time did not affect coating performance. The final chitosan concentration in WC2 was approximately 0.5% (*w*/*v*), with pH 5.6–6.0 at application.

Potato tubers were washed under running tap water, dried with tissue paper, and divided into three groups:

Control (CN): no coating applied.

WC1 treatment: tubers dipped in WC1 for 30 s and air-dried.

WC2 treatment: tubers dipped in WC2 for 30 min and air-dried.

Each group (100 g per treatment) was stored under three different conditions:

Room temperature (RT) (24 °C, 55% relative humidity);

Refrigeration (RF) (4 °C);

Incubator (IC) (20 °C, 74% relative humidity).

Storage was monitored for 42 days with measurements taken at 8-day intervals, corresponding to storage periods SP0 (0 days), SP1 (8 days), SP2 (16 days), SP3 (24 days), SP4 (32 days), SP5 (40 days), and SP6 (42 days).

### 2.3. Physicochemical Analysis

#### 2.3.1. Dry Matter%

In brief, 100 g of cut potatoes (wet weight) were dried at 70 °C for 48 h, and DM content was calculated as:

DM (%) = Dry weight/Wet weight × 100(1)

#### 2.3.2. Firmness

FR was measured using a digital fruit hardness tester (GY-4, Hangzhou, China). Readings were taken on both sides of peeled potato samples, and the average was recorded.

#### 2.3.3. Total Soluble Solids

TSS were determined with a digital refractometer (Atago PAL-1, Tokyo, Japan). 5 g of potato were homogenized with 50 mL of distilled water, filtered, and a few drops of the filtrate placed on the lens. Results expressed in °Brix were calculated as:

TSS (°Brix) =Refractometer reading × Dilution factor(2)

where refractometer reading refers to the value obtained from the instrument in °Brix and dilution factor refers to any dilution made to the sample.

DM, FR, and TSS were determined at each SP (SP0–SP6).

### 2.4. Statistical Analysis

All statistical analyses were conducted using SAS software [23] (version 9.4, SAS Institute Inc., Cary, NC, USA). A completely randomized design (CRD) was used to evaluate the effects of treatment (TRT), storage condition (SC), and storage period (SP), as well as their interactions, on dry matter (DM), firmness (FR), and total soluble solids (TSS) of potato samples. Each analysis was performed independently for each parameter, and all measurements were carried out in triplicate. The following linear model was adopted:

$$Y_{ijkl}=\mu +TRT_{i}+SC_{j}+SP_{k}+(TRT\times SC{)}_{ij}+(TRT\times SP{)}_{ik}+(SC\times SP{)}_{jk}+e_{ijkl}$$

where $Y_{ijkl}$ represents the observation of DM, FR, or TSS for the $i^{th}$ treatment, $j^{th}$ storage condition, and $k^{th}$ storage period; μ = overall mean; $TRT_{i}$ = effect of coating treatments (CN = uncoated control; WC1 = WPC; WC2 = WPC–chitosan); $SC_{j}$ = effect of storage conditions (RT = 24 °C, 55% RH; RF = 4 °C; IC = 20 °C ± 2 °C, 74% RH); $SP_{k}$ = effect of storage periods (SP0, SP1, … SP6 corresponding to 0–48 days at 8-day intervals); and $e_{ijkl}$ = random error term assumed to be normally distributed with mean zero and variance $\sigma_{e}^{2}$. Significant differences among means were determined using Duncan’s multiple range test [23] at *p* ≤ 0.05, and results were expressed as mean ± standard deviation (SD). Partial correlation analysis was also performed to examine the relationships among DM, FR, and TSS under different treatments, storage conditions, and storage periods, and to determine the significance of these associations.

## 3. Results

### 3.1. Effect of Treatments, Storage Conditions, and Storage Periods on Potato Quality Parameters

#### 3.1.1. Effect of Treatments

Table 1 shows the effect of treatments on the DM, FR, and TSS of potatoes. Application of edible coatings showed limited influence on DM content, as no significant (*p* > 0.05) differences were observed among treatments. The values ranged between 18.02–18.77%. FR exhibited a slight but non-significant (*p* > 0.05) improvement in coated samples compared with the CN. WC2 recorded the highest FR (7.63 N), but it did not differ significantly (*p* > 0.05) from CN (7.19 N) or WC1 (7.27 N). In contrast, TSS were significantly (*p* ≤ 0.05) affected by treatments. The CN (4.85 °Brix) and WC1 (4.78 °Brix) did not differ, while WC2 showed a small but significant (*p* ≤ 0.05) reduction in TSS (4.41 °Brix). These results indicate that whey protein coatings, especially with chitosan and additives (WC2), may slightly enhance FR but tend to lower soluble solid content without affecting DM.

#### 3.1.2. Effect of Storage Conditions

Table 2 shows the effect of storage conditions on the quality attributes of potatoes. DM values were not significantly (*p* > 0.05) different among storage conditions, with means of 18.10% (RT), 18.81% (RF), and 18.31% (IC). FR was also unaffected by storage conditions, ranging between 7.20–7.63 N without significant differences (*p* > 0.05).

In contrast, TSS were significantly (*p* ≤ 0.05) influenced by storage conditions. RF maintained the highest TSS value (5.67 °Brix), followed by RT (4.41 °Brix), while the IC condition led to the lowest TSS (3.95 °Brix). These findings suggest that although storage conditions did not alter DM or FR, RF was more effective in preserving soluble solids, whereas higher humidity and temperature (IC) accelerated the decline in TSS.

#### 3.1.3. Effect of Storage Periods

Table 3 shows the effect of SP (0–48 days) on DM, FR, and TSS of potatoes. DM content varied significantly (*p* ≤ 0.05) across SPs. The highest DM was recorded after 16 days (20.07%), while values at 0, 8, 24, 32, 40, and 48 days ranged between 17.70–18.51%, indicating a peak in DM after 16 days of storage followed by stabilization or decline. FR decreased progressively over time. Potatoes maintained high FR at 0, 8, 16, and 24 days (7.72–8.03 N), but FR dropped significantly (*p* ≤ 0.05) by 40 days (6.67 N) and reached the lowest value after 48 days (5.97 N), showing gradual softening with extended storage.

TSS was also affected by SP. Values increased up to 24–32 days, peaking at 5.06–5.07 °Brix, before declining at 40 days (4.58 °Brix) and reaching the lowest level at 48 days (4.36 °Brix). This trend indicates an initial accumulation of soluble sugars followed by degradation or utilization during prolonged storage.

These results demonstrate that SP strongly affected potato quality. DM peaked at 16 days before decreasing, FR declined progressively after 24–32 days, and TSS increased up to 24–32 days then dropped sharply by 48 days.

### 3.2. Interaction Effects of Storage Conditions and Edible Coatings on Potato Quality Parameters

#### 3.2.1. Dry Matter

**Figure 1:**
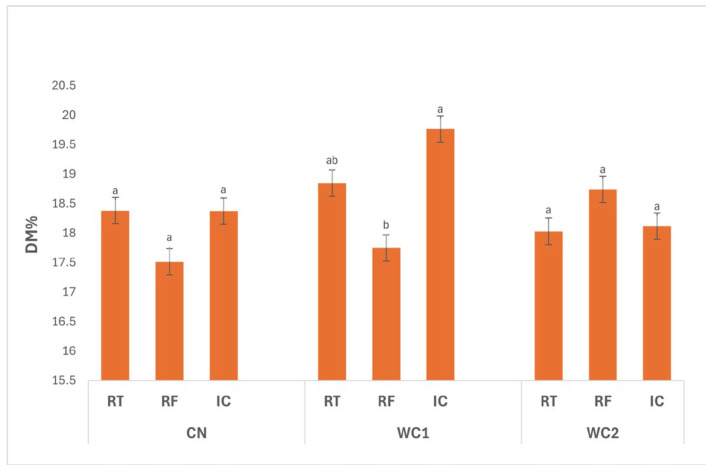
Effect of treatment × storage condition interaction on DM (%) of potatoes. Data are expressed as mean values (n = 3) ± standard deviation. Different letters above bars indicate significant differences (*p* ≤ 0.05) according to Duncan’s multiple range test.

The interaction between treatment and storage condition (Figure 1) showed a significant effect (*p* ≤ 0.05). A significant interaction (*p* ≤ 0.05) was observed between treatment and storage condition. In CN, DM did not differ significantly (*p* > 0.05) among storage conditions, with values of 18.38% (RT), 17.51% (RF), and 18.37% (IC), indicating that temperature had little influence on uncoated tubers.

In WC1, DM was significantly affected (*p* ≤ 0.05) by storage condition. The highest value was recorded under IC (19.76%), followed by RT (18.85%), while RF (17.75%) showed a significant reduction. This suggests that WC1 more effectively retained solids under IC, likely due to moderate surface dehydration and reduced water absorption, whereas refrigeration favored moisture gain, resulting in lower DM.

For WC2, no significant differences (*p* > 0.05) were observed among RT (18.03%), RF (18.74%), and IC (18.12%), indicating that this coating maintained stable DM levels across different storage conditions. Such stability reflects its balanced barrier properties, minimizing water migration regardless of temperature.

Overall, WC1 under IC exhibited the highest DM, confirming that this coating performed best under warmer storage by promoting controlled drying and preserving solids, while WC1 under RF showed the lowest DM due to higher moisture uptake at low temperature. These findings highlight that both coating formulation and storage environment significantly (*p* ≤ 0.05) influence moisture regulation and compositional stability in stored tubers.

#### 3.2.2. Firmness

**Figure 2:**
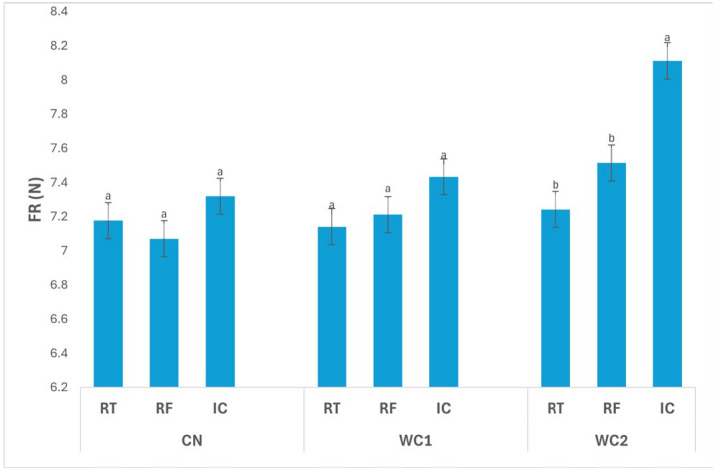
Effect of treatment × storage condition interaction on FR (N) of potatoes. Data are expressed as mean values (n = 3) ± standard deviation. Different letters above bars indicate significant differences (*p* ≤ 0.05) according to Duncan’s multiple range test.

The interaction between treatment and storage condition (Figure 2) had a significant effect (*p* ≤ 0.05) on FR. In CN, no significant differences (*p* > 0.05) were found among storage conditions, with values of 7.18 N (RT), 7.07 N (RF), and 7.32 N (IC), indicating that temperature alone did not markedly affect the firmness of uncoated tubers.

In WC1, FR also did not differ significantly (*p* > 0.05) among RT (7.14 N), RF (7.21 N), and IC (7.43 N), suggesting that this coating maintained consistent texture regardless of storage condition. The stable behavior of WC1 implies effective structural protection and minimized softening during storage.

In contrast, WC2 exhibited a significant difference (*p* ≤ 0.05) among storage conditions. The highest FR was recorded under IC (8.11 N), while RT (7.24 N) and RF (7.51 N) showed significantly lower values. These results indicate that WC2 was more effective in maintaining firmness under IC, likely due to reduced enzymatic activity and controlled moisture balance at the higher storage temperature.

Overall, WC2 under IC maintained the highest firmness, highlighting the synergistic effect of this coating and temperature condition in preserving tissue rigidity. Meanwhile, RT and RF samples showed lower FR values, reflecting mild softening associated with water redistribution or enzymatic degradation. These results demonstrate that coating formulation and storage environment significantly (*p* ≤ 0.05) influence the mechanical stability of stored tubers.

#### 3.2.3. Total Soluble Solids

**Figure 3:**
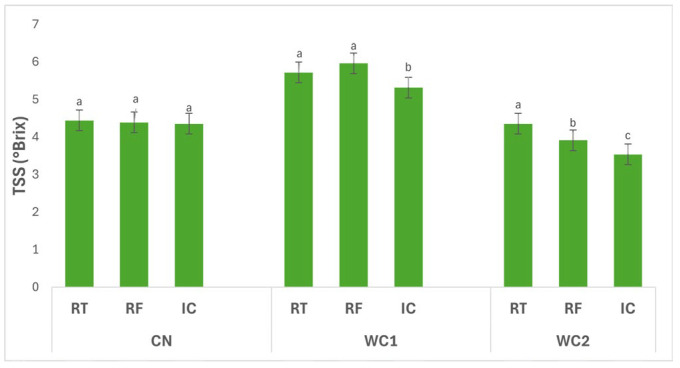
Effect of treatment × storage condition interaction on TSS (°Brix) of potatoes. Data are expressed as mean values (n = 3) ± standard deviation. Different letters above bars indicate significant differences (*p* ≤ 0.05) according to Duncan’s multiple range test.

Figure 3 illustrates the interaction effects of storage conditions (RT, RF, IC) and treatments (CN, WC1, WC2) on the TSS (°Brix) of potato tubers. The interaction between treatment and storage condition had a significant effect (*p* ≤ 0.05) on TSS. In CN, no significant differences (*p* > 0.05) were detected among RT (4.44 °Brix), RF (4.39 °Brix), and IC (4.35 °Brix), indicating that temperature did not influence the soluble solids content in uncoated tubers.

In WC1, TSS differed significantly (*p* ≤ 0.05) among storage conditions. The highest value was observed under RF (5.96 °Brix), followed by RT (5.72 °Brix), while IC (5.31 °Brix) showed a significant reduction. This pattern suggests that WC1 promoted greater accumulation or retention of soluble compounds under refrigeration, whereas higher temperature under IC led to a decrease, possibly due to enhanced metabolic utilization of sugars.

In WC2, TSS also varied significantly (*p* ≤ 0.05) with storage condition. The highest value was found under RT (4.35 °Brix), followed by RF (3.91 °Brix), while IC (3.53 °Brix) recorded the lowest. This trend indicates that WC2 was less effective in preserving soluble solids at higher temperature, where increased respiration or enzymatic activity likely depleted sugar content.

Overall, WC1 under RF maintained the highest TSS, reflecting favorable preservation of soluble compounds under low temperature, while WC2 under IC exhibited the lowest, emphasizing that both coating composition and storage environment significantly (*p* ≤ 0.05) affect sugar stability and postharvest metabolic behavior in stored tubers.

### 3.3. Interaction Effects of Treatments and Storage Periods on Potato Quality

#### 3.3.1. Dry Matter

**Figure 4:**
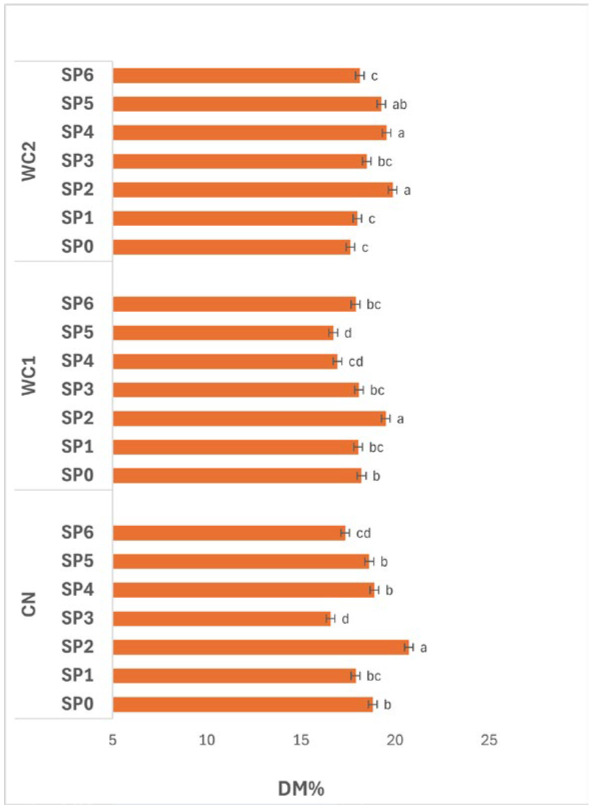
Effect of treatments and SP (0–48 days) on potato DM%. Data are expressed as mean values (n = 3) ± standard deviation. Different letters above bars indicate significant differences (*p* ≤ 0.05) according to Duncan’s multiple range test.

The interaction between treatment and SP (Figure 4) had a significant effect (*p* ≤ 0.05) on DM. In CN, DM changed noticeably over time, reaching its maximum at day 16 (SP2, 20.73%), which was significantly higher (*p* ≤ 0.05) than other periods. The lowest value occurred at day 24 (SP3, 16.58%), suggesting a sharp moisture uptake or solid loss at that stage. DM partially recovered thereafter, maintaining moderate levels between day 32–48 (SP4–SP6, 17.36–18.91%), indicating redistribution of water or slight re-drying in later stages.

In WC1, a similar time-dependent trend was observed. DM peaked significantly (*p* ≤ 0.05) at day 16 (SP2, 19.50%), followed by a gradual decline through day 24–40 (SP3–SP5, 16.72–18.09%), and a slight increase by day 48 (SP6, 17.90%). These results suggest that WC1 effectively retained solids during the early SP but became less efficient with extended storage, likely due to gradual weakening of the coating matrix or reduced moisture barrier integrity over time.

In WC2, significant differences (*p* ≤ 0.05) were also observed across SP. DM increased from day 0–16 (SP0–SP2, 17.63–19.86%), indicating controlled dehydration in early stages, and remained relatively high until day 32 (SP4, 19.54%) before slightly decreasing toward day 48 (SP6, 18.13%). This trend reflects the good stability of WC2 in maintaining solids throughout storage, particularly during mid-periods when moisture migration was more likely.

Overall, DM fluctuated significantly (*p* ≤ 0.05) throughout the 48-day SP, with day 16 (SP2) showing the highest values across all treatments. These results demonstrate that solid concentration typically peaks after two weeks of storage, after which gradual moisture gain occurs. Among coatings, WC2 maintained more consistent DM over time, indicating superior water-barrier performance and enhanced control of dehydration dynamics during prolonged storage.

#### 3.3.2. Firmness

**Figure 5:**
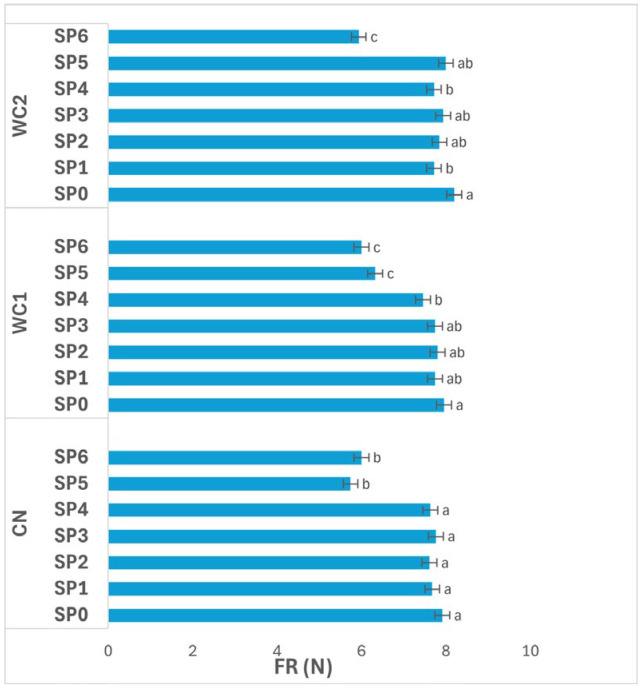
Effect of treatments and SP (0–48 days) on potato FR (N). Data are expressed as mean values (n = 3) ± standard deviation. Different letters above bars indicate significant differences (*p* ≤ 0.05) according to Duncan’s multiple range test.

Figure 5 shows the effect of treatments and SPs (0–48 days) on potato FR (N). The interaction between treatment and SP had a significant effect (*p* ≤ 0.05) on FR. In CN, FR remained statistically unchanged (*p* > 0.05) from day 0 to day 32 (SP0–SP4, 7.63–7.91 N), after which a marked decline occurred at day 40 and day 48 (SP5–SP6, 5.74–6.00 N). This reduction indicates progressive softening of uncoated tubers during the later storage stages, most likely due to tissue degradation and loss of turgor associated with moisture redistribution and enzymatic activity.

In WC1, FR showed a time-dependent decline that became significant (*p* ≤ 0.05) after day 32. Values remained relatively stable up to day 24 (SP3, 7.74 N), followed by a noticeable decrease at day 32 (7.46 N) and a sharp drop by day 40 and day 48 (SP5–SP6, 6.33–6.00 N). The gradual reduction suggests that although WC1 delayed softening during the early storage phase, its protective ability diminished over time, likely due to coating relaxation or structural weakening.

In WC2, FR also changed significantly (*p* ≤ 0.05) across storage. The highest FR was recorded at day 0 (SP0, 8.20 N), followed by stable values between day 8–32 (SP1–SP4, 7.72–7.93 N). FR remained high at day 40 (SP5, 8.00 N) but decreased sharply at day 48 (SP6, 5.93 N). This pattern suggests that WC2 effectively preserved texture through most of the storage duration, maintaining structural rigidity until the final period, after which softening became evident.

Overall, FR decreased progressively with time in all treatments, particularly after day 32 (SP4). However, WC2 maintained higher FR values over the 48-day period compared to CN and WC1, confirming its superior ability to reduce tissue softening and preserve mechanical integrity during extended storage.

#### 3.3.3. Total Soluble Solids

**Figure 6:**
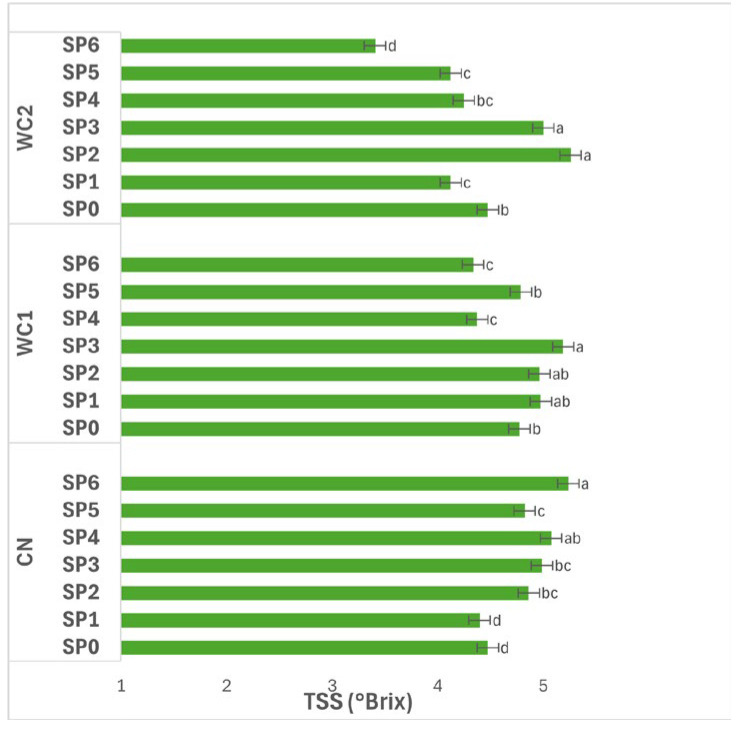
Effect of treatments and SP (0–48 days) on potato TSS (°Brix). Data are expressed as mean values (n = 3) ± standard deviation. Different letters above bars indicate significant differences (*p* ≤ 0.05) according to Duncan’s multiple range test.

Figure 6 illustrates the interaction effects of SP and treatments on TSS (°Brix) in potatoes. The interaction between treatment and SP had a significant effect (*p* ≤ 0.05) on TSS. In CN, TSS increased progressively over time, reaching its highest value at day 48 (SP6, 5.24 °Brix), which was significantly higher (*p* ≤ 0.05) than earlier periods. The lowest values were observed at day 0–8 (SP0–SP1, 4.40–4.47 °Brix), followed by gradual increases through day 16–40 (SP2–SP5, 4.82–5.08 °Brix). This steady rise suggests an accumulation of soluble compounds as storage progressed, possibly due to starch hydrolysis and concentration effects from water loss.

In WC1, TSS also varied significantly (*p* ≤ 0.05) during storage. Values increased up to day 24 (SP3, 5.19 °Brix), then declined notably at day 32 and day 48 (SP4–SP6, 4.37–4.34 °Brix). The initial increase indicates active solute formation or concentration during the early phase, while the subsequent decline reflects potential sugar utilization in respiration or leaching during extended storage.

In WC2, significant differences (*p* ≤ 0.05) were also recorded among SP. TSS peaked at day 16–24 (SP2–SP3, 5.26–5.00 °Brix), followed by a sharp decline at day 48 (SP6, 3.41 °Brix). The early rise followed by later reduction suggests that WC2 promoted early solute accumulation but could not prevent degradation or dilution at prolonged storage.

Overall, TSS fluctuated significantly (*p* ≤ 0.05) throughout the 48-day SP, with most treatments showing maximum values around day 16–24 and reductions thereafter. WC1 maintained relatively stable values through mid-storage, while WC2 displayed greater variation, indicating that coating type and storage duration jointly affected solute dynamics and sugar stability in the stored tubers.

### 3.4. Interaction Effects of Storage Periods and Storage Conditions on Potato Quality

#### 3.4.1. Dry Matter

**Figure 7:**
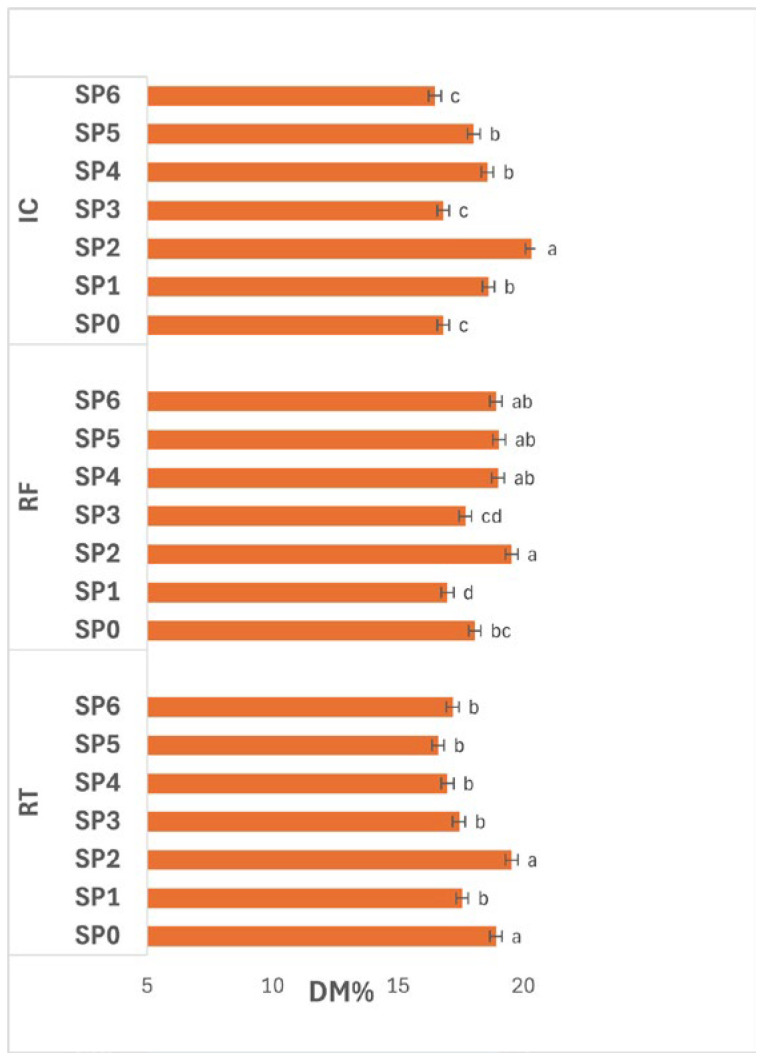
DM% of potatoes as affected by SP and storage conditions. Data are expressed as mean values (n = 3) ± standard deviation. Different letters above bars indicate significant differences (*p* ≤ 0.05) according to Duncan’s multiple range test.

Figure 7 illustrates the effect of SPs under different storage conditions on potato DM%. The interaction between storage condition and SP had a significant effect (*p* ≤ 0.05) on DM. Under RT, DM increased at SP2 (19.54%), which was significantly higher (*p* ≤ 0.05) than the other SPs. Values decreased afterwards, remaining between 16.60–17.56% (SP1, SP3–SP6), indicating moisture gain or partial solute loss as storage progressed.

Under RF, DM also varied significantly (*p* ≤ 0.05) across SP. The highest value was recorded at SP2 (19.54%), followed closely by SP4–SP6 (18.91–19.03%), while the lowest occurred at SP1 (16.97%). The overall pattern suggests that DM increased after mid-storage and remained relatively stable thereafter, reflecting improved water retention and reduced transpiration under low-temperature storage.

Under IC, DM differed significantly (*p* ≤ 0.05) among SPs, with the highest value at SP2 (20.34%) and the lowest at SP0, SP3, and SP6 (16.47–16.81%). Intermediate values were observed at SP1, SP4, and SP5 (18.02–18.61%), indicating that DM peaked mid-storage before declining again, possibly due to enhanced metabolic activity, respiration, or water absorption under the warmer IC condition.

Overall, DM showed clear temporal fluctuations (*p* ≤ 0.05) throughout storage across all conditions, with SP2 consistently representing the point of maximum solids concentration. This trend indicates that tubers initially underwent moderate dehydration followed by partial rehydration or metabolic water gain in later stages. The increase at SP2 may reflect the concentration of solids as free water was lost, whereas the subsequent decline likely resulted from enzymatic conversion of solids or water reabsorption. Among the tested storage conditions, RF maintained the most stable DM profile, suggesting it effectively balanced moisture retention and prevented excessive dehydration, thereby preserving tuber compositional stability during extended storage.

#### 3.4.2. Firmness

**Figure 8:**
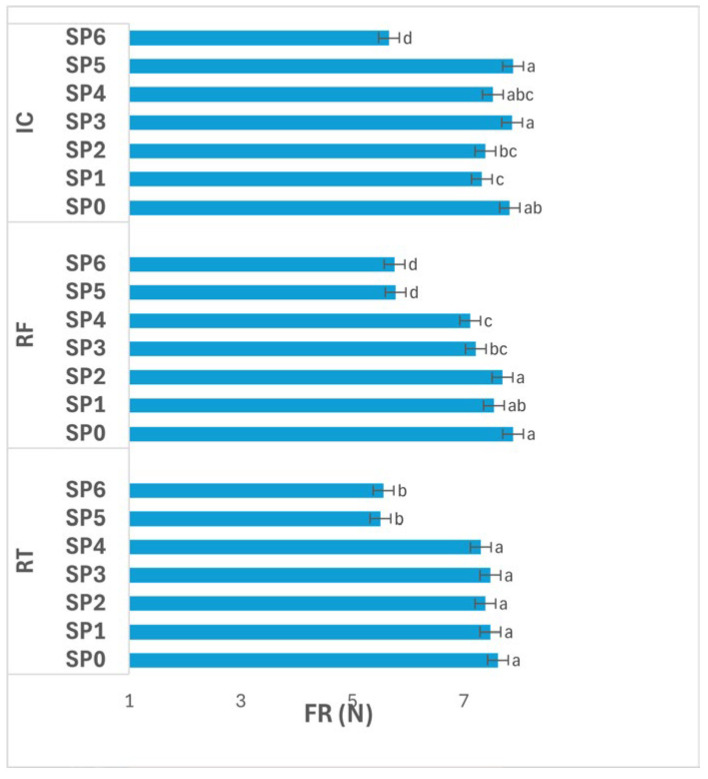
FR (N) of potatoes as affected by SP and storage conditions. Data are expressed as mean values (n = 3) ± standard deviation. Different letters above bars indicate significant differences (*p* ≤ 0.05) according to Duncan’s multiple range test.

Figure 8 presents the variation in potato FR (N) across storage periods (SPs; 0–48 days) under different storage conditions. The interaction between storage condition and SP had a significant effect (*p* ≤ 0.05) on FR.

Under RT, FR remained not significantly different (*p* > 0.05) between SP0–SP4 (7.31–7.61 N), followed by a significant decrease (*p* ≤ 0.05) at SP5–SP6 (5.50–5.56 N). This pattern indicates that tubers preserved their texture during the first 32 days, but experienced softening afterward, likely due to cell wall weakening, enzymatic depolymerization of pectic substances, and loss of turgor with advancing storage.

Under RF, FR values declined progressively from 7.88 N at SP0 to 5.75 N at SP6, with significant differences (*p* ≤ 0.05) observed after SP3. Although intermediate values showed gradual rather than abrupt drops, the continuous reduction reflects steady structural degradation, possibly linked to slow enzymatic softening and partial pectin solubilization under cold-induced stress.

Under IC, significant differences (*p* ≤ 0.05) were also evident among SPs. FR fluctuated between 7.32–7.88 N (SP1–SP5), with the highest readings at SP3 and SP5 (7.86–7.88 N), followed by a marked decline (*p* ≤ 0.05) to 5.66 N at SP6. The relatively stable FR through mid-storage suggests effective maintenance of cellular rigidity at moderate humidity and temperature, while the final drop reflects enhanced metabolic and enzymatic activity toward the end of storage, leading to texture loss.

Overall, FR decreased significantly (*p* ≤ 0.05) with advancing SP under all storage conditions, particularly after SP4. The general trend shows minimal softening during the first month, followed by pronounced texture loss during the last two weeks. Among the conditions, IC maintained higher FR values until late storage, suggesting better structural preservation, whereas RF induced gradual and consistent softening, and RT showed the most pronounced decline. These findings highlight that both temperature and duration strongly influence textural integrity, with late-stage degradation resulting from progressive enzymatic and structural weakening of tuber tissues.

#### 3.4.3. Total Soluble Solids

**Figure 9:**
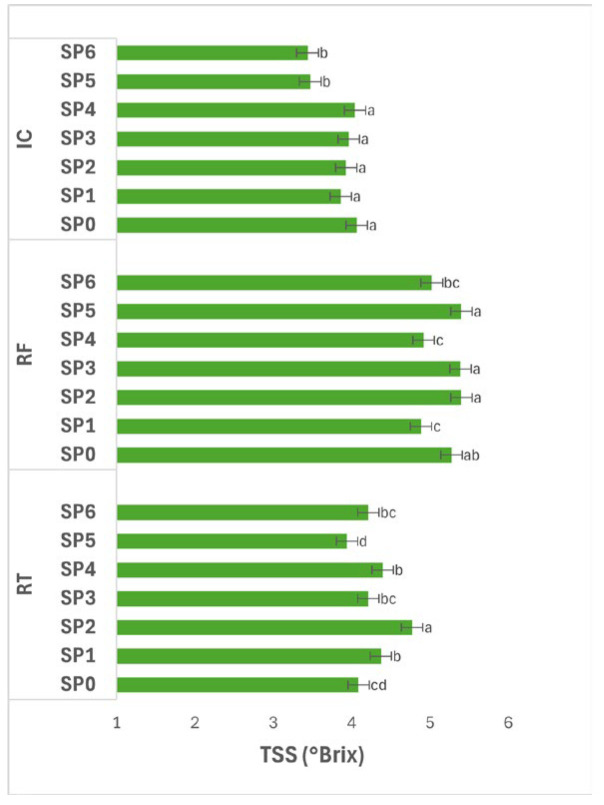
TSS (°Brix) of potatoes as affected by SP and storage conditions. Data are expressed as mean values (n = 3) ± standard deviation. Different letters above bars indicate significant differences (*p* ≤ 0.05) according to Duncan’s multiple range test.

Figure 9 demonstrates the effect of SPs (0–48 days) within different storage conditions on TSS (°Brix) of potatoes. The interaction between storage condition and SP had a significant effect (*p* ≤ 0.05) on TSS. Under RT, TSS fluctuated over time, reaching its highest value at SP2 (4.77 °Brix), which was significantly higher (*p* ≤ 0.05) than all other SPs. Moderate values were maintained at SP1, SP3, SP4, and SP6 (4.21–4.40 °Brix), while the lowest occurred at SP5 (3.94 °Brix). These changes indicate an initial increase due to solute concentration from moisture loss, followed by a gradual decline likely resulting from the metabolic consumption of soluble sugars.

Under RF, TSS also varied significantly (*p* ≤ 0.05) among SPs. Higher values were observed at SP2, SP3, and SP5 (5.39–5.40 °Brix), while lower values occurred at SP1 and SP4 (4.89–4.92 °Brix). The consistent maintenance of high TSS during mid- and late storage suggests that RF effectively reduced metabolic activity and water loss, thereby stabilizing soluble solids through the storage period.

Under IC, TSS remained statistically unchanged (*p* > 0.05) from SP0–SP4 (3.86–4.07 °Brix), then decreased significantly (*p* ≤ 0.05) at SP5–SP6 (3.44–3.47 °Brix). The decline toward the end of storage suggests enhanced respiration or enzymatic utilization of soluble sugars at elevated temperature, leading to depletion of soluble compounds.

Overall, TSS exhibited significant variation (*p* ≤ 0.05) across SP under all conditions, with SP2 generally showing peak values across treatments. The increase during early to mid-storage may be attributed to concentration effects and partial starch breakdown, whereas the later decrease reflects sugar consumption and metabolic conversion. Among storage conditions, RF maintained the highest and most stable TSS, indicating its efficiency in preserving soluble solids by minimizing respiration and moisture loss compared with RT and IC.

### 3.5. Correlation Analysis of Quality Parameters

Table 4 presents the partial correlation coefficients among DM, FR, and TSS of potatoes under different treatments (CN, WC1, WC2) and storage conditions (RT, RF, IC). The coefficients reveal weak and inconsistent relationships, highlighting that the three quality parameters do not follow a uniform trend across treatments or storage environments.

For DM–FR, positive correlations were observed under CN (0.083), WC1 (0.191), and RT (0.304), while WC2 (−0.099), RF (−0.117), and IC (−0.008) showed negative or near-zero values. This indicates that in some cases higher DM coincides with slightly firmer tubers, whereas under other conditions DM and FR vary independently. For DM–TSS, the coefficients ranged from negative in CN (−0.119), RF (−0.096), and IC (−0.032) to positive in WC1 (0.026), WC2 (0.264), and RT (0.129). This suggests that DM and TSS may occasionally follow the same trend, particularly in WC2 and RT, but diverge under RF or IC storage.

For FR–TSS, the associations also varied, being negative under CN (−0.171), RF (−0.037), and IC (−0.047), but slightly positive in WC1 (0.026), WC2 (0.014), and RT (0.242). This reflects that firmer potatoes sometimes retained more soluble solids, especially at RT, but this was not consistent across treatments.

Overall, the table suggests that DM, FR, and TSS respond differently to coating treatments and storage conditions. Each trait is likely governed by distinct physiological mechanisms—DM by starch content, FR by cell wall integrity, and TSS by sugar metabolism—resulting in limited interdependence among them.

Table 5 presents the partial correlation coefficients among DM, FR, and TSS of potatoes across different SPs (0, 8, 16, 24, 32, 40, and 48 days). The coefficients reveal fluctuating relationships, indicating that the three quality parameters vary independently over time and are influenced by distinct physiological processes during storage.

For DM–FR, the associations shifted from negative at day 0 (−0.276) and day 32 (−0.253) to weakly positive at days 16 (0.209), 24 (0.028), and 48 (0.065). These fluctuations suggest that DM and FR did not follow a consistent pattern, with periods of alignment followed by divergence as storage progressed.

For DM–TSS, the coefficients also varied, showing weak negative values at days 0 (−0.052), 8 (−0.165), 32 (−0.068), 40 (0.058), and 48 (0.055), but stronger positive relationships at day 24 (0.392). This indicates that increases in DM occasionally coincided with higher soluble solids, though the relationship was not stable across storage.

For FR–TSS, the associations were mostly weak but alternated between positive and negative across periods. Notably, day 16 (0.345) and day 24 (0.392) showed positive relationships, suggesting firmer tubers tended to retain more soluble solids at these times, whereas a strong negative value was observed at day 32 (−0.623), pointing to a divergence between FR and soluble solids beyond one month of storage.

Taken together, the results demonstrate that DM, FR, and TSS respond differently as storage progresses, with no consistent interdependence. Instead, each parameter appears to be influenced by separate physiological mechanisms—such as starch hydrolysis, cell wall softening, and sugar accumulation—that vary in intensity at different SPs.

## 4. Discussion

The present study demonstrated that the physicochemical responses of potatoes—expressed through DM, FR, and TSS—were significantly affected by coating composition, storage condition, and storage duration (SP). These findings confirm that the interaction between edible polymeric films and tuber physiology determines the extent of postharvest preservation. While DM was relatively stable, both FR and TSS were more responsive to coating formulation and storage, reflecting the dynamic balance between dehydration, enzymatic hydrolysis, and respiration during storage. The results emphasize that the effectiveness of chitosan- and whey protein-based coatings lies not only in their ability to form physical barriers but also in their intrinsic physicochemical characteristics, such as hydrophilicity, charge density, and intermolecular bonding capacity, which govern permeability and film durability.

In general, the small variation in DM among treatments (Figure 1) indicates that the coatings primarily influenced surface water transfer rather than internal solids distribution. WC2, composed of whey protein and chitosan, exhibited higher DM stability, suggesting reduced moisture exchange due to improved barrier formation. The interaction between the hydrophilic amino and hydroxyl groups of chitosan and the protein backbone of whey protein likely enhanced cohesion and cross-link density, producing a denser network with lower water-vapor transmission. Similar behavior was reported by Xin et al. [24] in whey protein-based coatings for fresh-cut apples, where cross-linked protein networks suppressed water migration and oxidative browning. The physicochemical principles underlying this behavior are well-established: proteins such as β-lactoglobulin and α-lactalbumin exhibit compact secondary structures stabilized by disulfide bonds and hydrogen interactions, providing excellent oxygen and aroma barriers [7,10]. When blended with chitosan, electrostatic attraction between negatively charged carboxyl groups in proteins and positively charged amino groups in chitosan forms a semi-interpenetrating network, which significantly enhances mechanical strength and water resistance [8]. The stability of DM under these conditions reflects not only restricted vapor diffusion but also suppressed transpiration and water redistribution inside the tuber, consistent with earlier findings showing that edible films reduce weight loss and dehydration without altering solids concentration [25,26].

The observed differences in FR (Figure 3) further demonstrate the functional contribution of the coating’s physicochemical structure to texture retention. WC2 preserved FR more effectively than WC1, indicating that the composite formulation provided a stronger structural barrier that mitigated enzymatic softening. The chitosan–protein blend likely created a film with greater tensile strength, higher crystallinity, and controlled porosity, reducing gas and moisture transfer. This is consistent with the work of Botalo et al. [27], who found that alginate–whey protein films with antioxidant additives exhibited higher UV stability, mechanical integrity, and reduced enzymatic degradation. Chitosan’s semi-crystalline nature and hydrogen-bonding capacity contribute to these advantages by forming a continuous matrix capable of retaining shape under humidity fluctuations [8]. Moreover, chitosan carries protonated amino groups (–NH_3_^+^) under mildly acidic conditions, imparting cationic surface charge that can interact with negatively charged cell membranes, providing antimicrobial activity and preserving FR. This charge-dependent behavior has been observed in other food systems; for example, Chit et al. [13] reported that chitosan–alginate coatings reduced softening and microbial decay in purple sweet potatoes during storage. In the present study, the improved FR of WC2 likely reflects both this antimicrobial effect and reduced cell-wall degradation due to lower oxygen availability within the coating barrier. The combination of whey protein and chitosan thus produced a cohesive film with complementary functional roles: whey protein provided elasticity and reduced oxygen permeability, while chitosan supplied rigidity and antimicrobial protection.

Changes in TSS (Figure 3) were more pronounced, confirming that sugar metabolism is the most sensitive indicator of coating efficiency. The early increase in TSS during initial storage likely resulted from starch hydrolysis, whereas the subsequent decrease after approximately 32 days (SP4) reflected consumption of soluble sugars through respiration. These fluctuations were moderated in coated tubers, particularly under WC2, demonstrating that the film’s gas barrier reduced oxygen entry and delayed respiratory metabolism. Such metabolic moderation aligns with findings by Tarawneh et al. [28], who observed that whey protein coatings on apples slowed sugar accumulation and prevented oxidative stress during cold storage. The physicochemical explanation for this lies in the semi-permeable nature of biopolymer films: by lowering oxygen diffusion and increasing internal CO_2_ concentration the coating restricts oxidative enzymes and sugar degradation [11,29]. This mechanism corresponds with the high hydrophilicity of chitosan, which allows limited water mobility but significantly restricts non-polar gases. However, as storage progresses, absorbed water plasticizes the film, increasing its permeability [8]. This explains the decline in TSS and FR after SP4, when film relaxation and microcracking likely reduced the barrier function.

The influence of storage conditions further clarifies these mechanisms. Potatoes stored under RF exhibited higher TSS stability and slower FR decline compared with those at RT or IC (Table 2; Figure 7, Figure 8 and Figure 9). Low temperature reduces vapor pressure gradients, suppresses enzymatic activity, and prevents coating deformation. In contrast, under IC, elevated temperature accelerated molecular motion, increasing polymer free volume and thereby permeability. This observation is consistent with the findings of Ngo et al. [12], who reported that pectin/nano-chitosan coatings better preserved FR and TSS at RF compared with ambient storage. The progressive changes in DM, FR, and TSS across SP (Figure 6, Figure 7 and Figure 8) reflect this interplay between environmental stress and film behavior. DM peaked around SP2 (day 16) and then declined, indicating gradual dehydration, whereas FR remained stable until SP4 (day 32) before decreasing, and TSS dropped sharply after SP4, marking accelerated respiration. The synchronized drop in TSS and FR implies that both textural and metabolic degradation intensified once the coating’s cohesive network lost integrity. As reported by Elsabee and Abdou [8], water absorption competes with inter-chain hydrogen bonding in chitosan–protein films, leading to swelling, lower tensile strength, and higher gas permeability. Therefore, the observed loss of barrier efficiency after one month likely represents polymer plasticization rather than chemical instability.

The correlation analysis (Table 4 and Table 5) supports this interpretation. Weak correlations among DM, FR, and TSS indicate independent physiological pathways for water balance, structural firmness, and sugar metabolism, although transient correlation between FR and TSS appeared at SP4, reflecting the simultaneous onset of softening and sugar depletion. Similar relationships were observed by Abbasi et al. [30] and Xiao et al. [31], who linked firmness loss and starch-to-sugar conversion during prolonged storage, and by Rosales et al. [32], who found that elevated sugar content coincided with reduced viscosity and increased antioxidant activity in aging tubers. These processes stem from respiration-driven starch breakdown accompanied by CO_2_ and H_2_O release, leading to reductions in DM and FR. Coatings delay these effects by limiting oxygen diffusion and enzyme activity [6,25,26,33], but the delay is finite because polymer hydration gradually compromises film compactness.

Collectively, the results demonstrate that coating performance depends not only on chemical composition but also on molecular interactions between the polymers and their environment. The synergistic behavior of whey protein and chitosan explains why WC2 outperformed WC1, maintaining FR and moderating TSS for approximately one month. This outcome aligns with previous research indicating that composite coatings outperform single biopolymer films because of enhanced intermolecular bonding and microstructural compatibility [8,29]. The superior performance of WC2 can be attributed to the balance between hydrophilic and hydrophobic domains, creating a dynamic yet cohesive matrix that adapts to humidity without immediate structural failure. However, beyond SP4, film plasticization and phase separation likely occurred, reducing barrier efficacy. To extend shelf life beyond 32 days, future research should target molecular reinforcement strategies such as enzyme-induced cross-linking, covalent grafting of hydrophobic agents, or multilayer composite designs. These approaches have been proposed for extending stability in protein–polysaccharide films [24,27]. Such modifications would help maintain low gas permeability and delay polymer relaxation during extended storage, improving the long-term physicochemical protection of tubers.

Overall, the present findings confirm that WC2 provided the most effective protection, primarily through the physicochemical synergy of whey protein and chitosan. Their complementary molecular properties—elasticity, cationic activity, and hydrophilic–hydrophobic balance—produced a semi-permeable, antimicrobial, and gas-resistant matrix that delayed physiological degradation. The decline in coating effectiveness after SP4 underscores the importance of understanding polymer relaxation and water–film interactions. Overall, the study highlights how edible coatings can preserve potato quality through controlled mass transfer and mechanical stabilization, and how these effects are governed by the fundamental physicochemical nature of the coating polymers rather than by their composition alone.

## 5. Conclusions

This study demonstrated that whey protein-based edible coatings, particularly the whey protein–chitosan formulation (WC2), effectively preserved the postharvest quality of potato tubers during storage under varying environmental conditions. The application of WC2 enhanced FR retention and delayed deterioration compared with both the CN and WC1. Among the examined factors, SP exerted the most pronounced influence on potato quality attributes. DM increased during the initial stages of storage (up to 16–24 days) before declining, FR progressively decreased with extended storage, and TSS exhibited a marked reduction beyond 32 days.

Storage conditions further modulated these effects, with RF emerging as the most favorable environment for maintaining overall quality. RF effectively mitigated losses in soluble solids and slowed the rate of softening, whereas RT and IC conditions accelerated compositional and textural degradation. The interaction between coatings and storage environments revealed distinct performance trends—WC1 under IC conditions yielded higher DM stability, while WC2 under RF provided superior texture preservation—highlighting the role of both coating composition and storage temperature in influencing tuber physiology.

Correlation analysis confirmed that DM, FR, and TSS responded independently to coatings, storage conditions, and SP, reflecting their regulation by distinct physiological mechanisms associated with moisture balance, cellular integrity, and sugar metabolism. While transient associations appeared at certain stages, these relationships were not consistent across time, underscoring the dynamic and multifactorial nature of postharvest changes in potatoes.

Overall, the integration of whey protein–chitosan coatings with RF represents a promising, biodegradable approach for extending potato shelf life and maintaining quality. The findings support the broader use of biopolymer-based coatings as sustainable alternatives to conventional packaging, contributing to reduced postharvest losses and advancing environmentally responsible food preservation strategies.

## Figures and Tables

**Table 1:** Effect of treatments on potato quality parameters.

Treatment	Dry Matter (DM) (%) ^1^	Firmness (FR) (N)	Total Soluble Solids (TSS) (°Brix)
CN	18.43 ^a^ ± 0.36	7.19 ^a^ ± 0.14	4.85 ^a^ ± 0.15
WC1	18.02 ^a^ ± 0.34	7.27 ^a^ ± 0.13	4.78 ^a^ ± 0.17
WC2	18.77 ^a^ ± 0.37	7.63 ^a^ ± 0.26	4.41 ^b^ ± 0.18

^1^ Values are expressed as mean ± SE. Different superscript letters within a column indicate significant differences at *p* ≤ 0.05 (Duncan’s multiple range test).

**Table 2:** Effect of storage conditions on potato quality parameters.

Storage Condition	DM (%) ^1^	FR (N)	TSS (°Brix)
RT	18.10 ^a^ ± 0.30	7.20 ^a^ ± 0.13	4.41 ^b^ ± 0.09
RF	18.81 ^a^ ± 0.37	7.27 ^a^ ± 0.14	5.67 ^a^ ± 0.15
IC	18.31 ^a^ ± 0.39	7.63 ^a^ ± 0.25	3.95 ^c^ ± 0.13

^1^ Values are expressed as mean ± SE. Different superscript letters within a column indicate significant differences at *p* ≤ 0.05 (Duncan’s multiple range test).

**Table 3:** Effect of SP on potato quality parameters.

Storage Period (SP)	DM (%) ^1^	FR (N)	TSS (°Brix)
SP0	18.28 ^b^ ± 0.37	8.03 ^a^ ± 0.08	4.60 ^a^^b^ ± 0.17
SP1	18.14 ^b^ ± 0.45	7.72 ^a^ ± 0.07	4.51 ^b^ ± 0.15
SP2	20.07 ^a^ ± 0.68	7.76 ^a^ ± 0.08	5.06 ^a^ ± 0.27
SP3	17.70 ^b^ ± 0.43	7.81 ^a^ ± 0.10	5.07 ^a^ ± 0.31
SP4	18.51 ^b^ ± 0.57	7.59 ^a^ ± 0.09	4.59 ^a^^b^ ± 0.14
SP5	18.24 ^b^ ± 0.60	6.67 ^b^ ± 0.59	4.58 ^a^^b^ ± 0.33
SP6	17.89 ^b^ ± 0.55	5.97 ^c^ ± 0.10	4.36 ^b^ ± 0.33

^1^ Values are expressed as mean ± SE. Different superscript letters within a column indicate significant differences at *p* ≤ 0.05 (Duncan’s multiple range test).

**Table 4:** Partial correlation coefficients among DM, FR, and TSS of potatoes under different treatments and storage conditions.

Variables Compared	CN	WC1	WC2	RT	RF	IC
DM–FR	0.083 ^1^	0.191	−0.099	0.304	−0.117	−0.008
DM–TSS	−0.119	0.026	0.264	0.129	−0.096	−0.032
FR–TSS	−0.171	0.026	0.014	0.242	−0.037	−0.047

^1^ Values represent partial correlation coefficients; positive values indicate direct relationships, negative values indicate inverse relationships.

**Table 5:** Partial correlation coefficients among DM, FR, and TSS of potatoes at different SPs.

Variables Compared	SP0	SP1	SP2	SP3	SP4	SP5	SP6
DM–FR	−0.276	−0.008	0.209	0.028	−0.253	−0.047	0.065
DM–TSS	−0.052	−0.165	−0.068	0.140	0.058	0.078	0.055
FR–TSS	0.081	0.345	0.392	−0.623	−0.054	−0.329	0.093

Values represent partial correlation coefficients; positive values indicate direct relationships, negative values indicate inverse relationships.

## Data Availability

The original contributions presented in this study are included in the article. Further inquiries can be directed to the corresponding authors.

## Abbreviations

The following abbreviations are used in this manuscript:

CN	Control
WPC	whey protein concentrate
WC1	WPC coating
WC2	WPC–chitosan coating with additives
DM	Dry matter
FR	Firmness
TSS	Total soluble solids
RT	Room temperature
RF	Refrigeration
IC	Incubator
SP	Storage period
